# SOCS1 expression in cancer cells: potential roles in promoting antitumor immunity

**DOI:** 10.3389/fimmu.2024.1362224

**Published:** 2024-02-13

**Authors:** Subburaj Ilangumaran, Yirui Gui, Akhil Shukla, Sheela Ramanathan

**Affiliations:** Department of Immunology and Cell Biology, Faculty of Medicine and Health Sciences, Université de Sherbrooke, Sherbrooke, QC, Canada

**Keywords:** SOCS1, tumor suppressor, growth control, antigen presentation, tumor immunogenicity, checkpoint inhibition

## Abstract

Suppressor of cytokine signaling 1 (SOCS1) is a potent regulator immune cell responses and a proven tumor suppressor. Inhibition of SOCS1 in T cells can boost antitumor immunity, whereas its loss in tumor cells increases tumor aggressivity. Investigations into the tumor suppression mechanisms so far focused on tumor cell-intrinsic functions of SOCS1. However, it is possible that SOCS1 expression in tumor cells also regulate antitumor immune responses in a cell-extrinsic manner via direct and indirect mechanisms. Here, we discuss the evidence supporting the latter, and its implications for antitumor immunity.

## 1 SOCS1-dependent checkpoints in innate and adaptive immune responses

SOCS1 is a negative feedback regulator of cytokine-induced Janus kinase (JAK)-Signal transducer and activation of transcription (STAT) signaling pathway and is the founding member of the SOCS protein family that contains eight members, namely, SOCS1 to SOCS7 and CISH (1–4). All SOCS family proteins have a central Src homology 2 (SH2) domain and a conserved SOCS box at the carboxy terminus. The SH2 domain interacts with phosphorylated JAKs and other phospho-tyrosine containing proteins, whereas the SOCS box promotes ubiquitination of many SOCS1-binding proteins for subsequent degradation by proteasomes (5, 6). Even though SOCS1 was discovered as an inhibitor of IL-6 signaling, generation of SOCS1-deficient mice revealed its indispensable role in attenuating IFNγ signaling and consequent inflammatory responses (1–3, 7, 8). Reversal of IFNγ-driven perinatal lethality in SOCS1-deficient mice through ablation of the *Rag2* gene indicated that T lymphocytes are the major producer of IFNγ in these mice (7). SOCS1-dependent regulation of T cell activation is manifested predominantly in the CD8^+^ T cell compartment than in the CD4^+^ compartment (9–12). CD8^+^ T cells isolated from SOCS1-deficient mice display a memory-like phenotype and show increased sensitivity to the IL-2 family cytokines, especially IL-15 (11–15). T regulatory cells, which rely on IL-2 for survival and homeostasis, naturally downmodulate SOCS1 expression through constitutive expression of miR-155 that targets the *Socs1* transcript (16).

Aberrant activation of CD8^+^ T cells and their exuberant production of IFNγ in SOCS1-deficient mice could arise from the compound effect of (i) excess production of inflammatory cytokines from macrophages and dendritic cells that activate T cells, and (ii) increased cytokine-driven proliferation and consequent priming for antigen stimulation. SOCS1 is essential to control Toll-like receptor 4 (TLR4) signaling induced by bacterial lipopolysaccharides (LPS), and thus macrophages and dendritic cells from SOCS1 deficient mice show increased production of inflammatory cytokines (17–20). Some of the inflammatory cytokines such as IL-16 and IL-21 can synergize with cytokines that promote T cell homeostasis such as IL-15 and IL-7 to induce antigen non-specific activation of naïve CD8^+^ T cells that acquire increased sensitivity to autoantigens (14, 21). We have shown that CD8^+^ T cells from SOCS1 deficient mice can elicit autoinflammatory disease upon recognition of cognate autoantigens (21–23). SOCS1 deficiency in myeloid and lymphoid cells has been shown to increase susceptibility to experimental inflammatory and autoimmune diseases (24, 25). Moreover, haploinsufficiency for the *SOCS1* gene is associated with inflammatory syndrome and autoimmunity in human (26, 27).

The SOCS1-dependent checkpoint in T cells has been exploited to attenuate inflammatory diseases in experimental settings. A SOCS1-mimetic peptide Tkip, which resembles the peptide sequence surrounding the JAK-binding sequence of SOCS1, and similar peptides has been shown to inhibit inflammatory diseases such as experimental psoriasis and lung inflammation (28–35). In these settings, the SOCS1-mimetic peptide likely operates on both myeloid and T cells to attenuate their inflammatory responses. SOCS1-mimetic peptides can also impact parenchymal cells, as SOCS1 released from alveolar macrophages was reported to attenuate activation of airway epithelial cells (36).

SOCS1 can also modulate antitumor immunity in different ways depending on its expression level in antitumor lymphocytes and in tumor cells. It restrains the effector functions of tumor reactive CD8^+^ T cells as transduction of with *Socs1*-targeting microRNA miR-155 into these cells enhances their cytokine responsiveness resulting in improved efficiency to control tumors (37, 38). On the other hand, many lines of evidence suggest that SOCS1 expression in cancer cells can positively impact the development of antitumor immune responses, for which we present evidence in the following sections.

## 2 Cell-intrinsic tumor suppression mechanisms of SOCS1

Following the seminal finding that the *SOCS1* gene is repressed in hepatocellular carcinoma by promoter CpG methylation, similar epigenetic and miRNA-mediated SOCS1 loss has been reported in many cancers including, neuroblastoma, myeloid leukemias and colorectal, pancreatic, breast, prostate and ovarian cancers (24, 39–49). Genetic studies demonstrating the susceptibility of SOCS1-deficient mice to develop radiation-induced leukemias and to experimental induction of hepatocellular carcinoma and colorectal cancer confirm that SOCS1 is a *bona fide* tumor suppressor (50–54). The tumor suppressor function of SOCS1 can be attributed to various mechanisms, which may operate in various combinations in diverse cancers in a context-dependent manner. These include attenuation of cytokine- and growth factor- induced oncogenic signaling via JAK and receptor tyrosine kinases (RTK) (55–58), potentiation of p53-mediated tumor suppressor functions (59, 60), inhibition of the paradoxical oncogenic functions of tumor suppressors such as cyclin-dependent kinase inhibitor 1A (CDKN1A; commonly known as p21Cip1/WAF1) and NFE2 Like BZIP Transcription Factor 2 (NEF2L2; commonly known as Nuclear factor erythroid 2-related factor 2 or NRF2) (52, 53, 61, 62). Moreover, SOCS1 expressed in mesenchymal cells can inhibit tumor promoting inflammatory cytokine signaling that establishes a tumor-promoting microenvironment (51, 54, 63). The cell-intrinsic tumor suppressor functions of SOCS1 were mostly gleaned from studies using overexpressed SOCS1, as endogenous SOCS1 is induced following exposure to cytokines, growth factors and myriad of other stimuli, and its expression regulated at the transcriptional and post-translational level. Among the potential tumor suppressor mechanisms of SOCS1, only the regulation of the oncogenic function of p21 is genetically proven (53), and all other mechanisms remain to be tested.

## 3 Evidence for modulation of antitumor immunity by SOCS1 expressed in tumor cells

**Figure 1:**
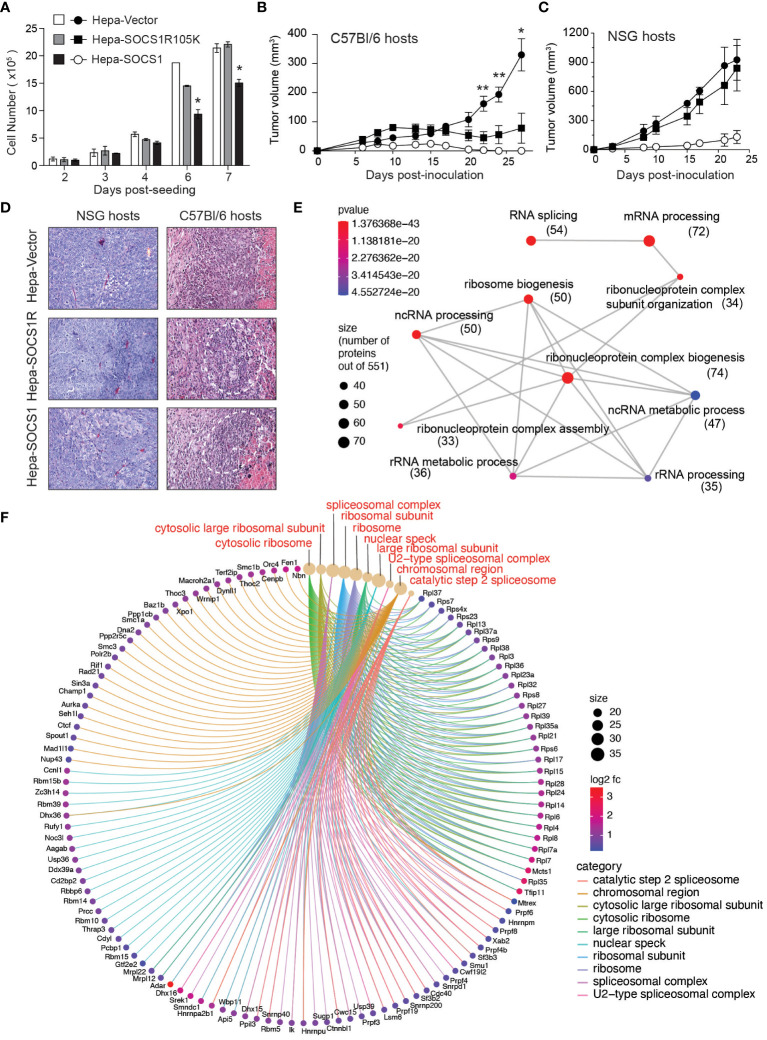
SOCS1R105K mutant unravels the potential role of SOCS1 in promoting antitumor immunity. **(A)** 1 ×10^5^ Hepa-Vector, Hepa-SOCS1 or Hepa-SOCS1R105K cells were plated on 100 mm Petri dishes in triplicates and cultured by replenishing medium every 2 days. Cells were trypsinized on the indicated days and counted. Representative data from one of the two experiments are shown (mean ± standard error of mean; *p <0.05, Student’s t-test). **(B, C)** 5 ×10^6^ Hepa-Vector, Hepa-SOCS1 or Hepa-SOCS1R105K cells were implanted subcutaneously in C57BL/6 **(B)** or NSG **(C)** mice (n=7-8) and tumor growth monitored as detailed in (56) Reference. Mean ± standard error of mean; *p <0.05, **p <0.01, Student’s t-test. **(D)** Representative hemoxylin and eosin-stained sections of tumors resected from NSG and C57BL/6 mice. In C57BL/6 hosts, all tumors had a central necrotic area and a reactive rim composed of infiltrating immune cells (encircles with dotted lines) that were more pronounced in Hepa-SOCS1 and Hepa-SOCS1R105K tumors than in Hepa-vector tumors. Data shown in **A–C** were generated from experiments approved by the Université de Sherbrooke Ethics Committee for Animal Care and Use. **(E, F)** Proteomes of Hepa-Vector and Hepa-SOCS1 cells were studied by tandem mass spectrometry, as detailed in (65) Reference. Proteins that were significantly upregulated in Hepa-SOCS1 cells (≥ 0.5 fold change and ≥1.3 -log10 p-Value; 551 proteins) were subjected to gene ontology analysis. Enrichment plot for biological pathways (emapplot, **E**) and cellular compartments (cnetplot, **F**) were made using the SRplot (https://www.bioinformatics.com.cn/en) data analysis tool. Number of proteins within the biological pathways are indicated in parenthesis. The mass spectrometry data used to generate **Figures 1E, F** are deposited to the ProteomeXchange Consortium via the PRIDE partner repository (https://www.ebi.ac.uk/pride/archive/) with the dataset identifier PXD047908.

The idea that SOCS1 expressed in tumor cells may influence the induction of antitumor immune response came from one of our unpublished observations. While studying the tumor suppressor functions of SOCS1, we compared the murine hepatocellular carcinoma cell line Hepa 1-6 expressing wildtype SOCS1 (Hepa-SOCS1), an SH2 domain mutant of SOCS1 (R105K) that does not inhibit IFNγ signaling (Hepa-SOCS1R) or a control vector (Hepa-Vector) (64) for their ability to form tumors. Consistent with the ability of SOCS1 to inhibit growth factor-induced RTK signaling, Hepa-SOCS1 cells showed appreciably reduced growth compared to Hepa-Vector or Hepa-SOCS1R cells *in vitro*, and this growth reduction was significant at higher cell densities (**Figure 1A**). Upon subcutaneous implantation in immuno-deficient NOD.*scid.gamma* (NSG) mice or immuno-competent C57BL/6 mice, Hepa-Vector cells formed large tumors, while Hepa-SOCS1 cells grew poorly in both hosts, consistent with the inhibition of mitogenic signals by SOCS1. On the other hand, Hepa-SOCS1R cells showed retarded growth in C57BL/6 mice but formed large tumors in NSG mice (**Figures 1B, C**). The possibility that the SOCS1R105K mutant might play a dominant negative role is unlikely because Hepa-SOCS1R cells did not grow more robustly than Hepa-Vector cells. Moreover, Hepa-SOCS1 and Hepa-SOCS1R tumors growing in C57BL/6 mice displayed more prominent inflammatory immune cell infiltrations adjacent to necrotic areas than Hepa-Vector tumors (**Figure 1D**). These data indicated that blocking the ability of SOCS1 to inhibit mitogenic cytokine and growth factor signaling using the R105K mutation has unmasked a hitherto unappreciated potential of SOCS1 in promoting antitumor immunity. We hypothesize that SOCS1 expressed in tumor cells could impact antitumor immune responses through multiple mechanisms that are discussed below.

## 4 Potential anti-tumor immune functions of SOCS1 expressed in tumor cells

### 4.1 Promoting tumor antigen processing and presentation

**Figure 2:**
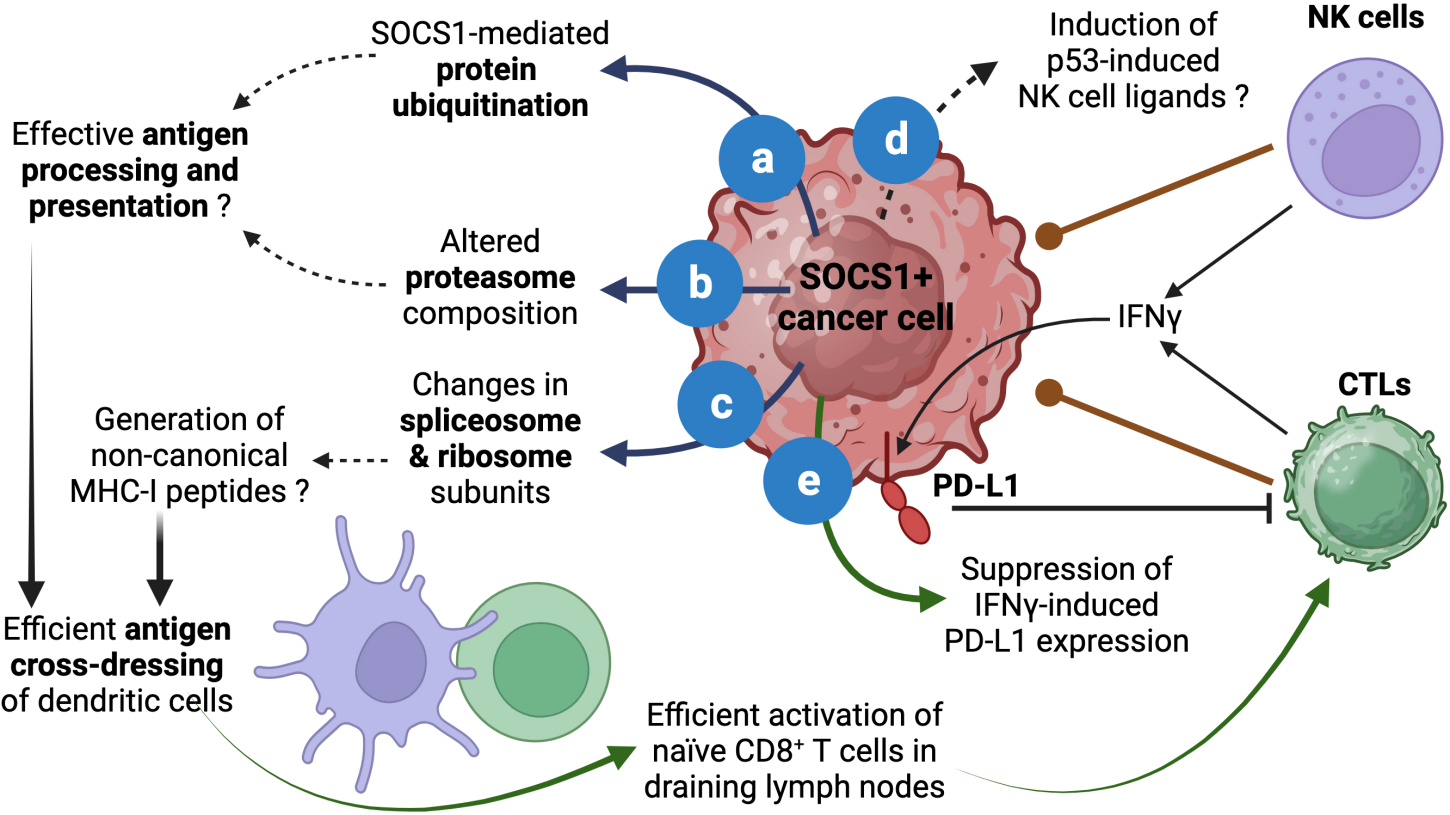
Proposed roles of SOCS1 in enhancing antitumor immunity. The role of SOCS1 in promoting protein ubiquitination and proteasomal degradation has been well documented **(A)**. Stable SOCS1 expression in cancer cells alters proteosome composition **(B)**. Both **(A, B)** can modulate the generation of antigenic peptides for loading MHC-I. SOCS1 expression also modulates spliceosome and ribosome subunits **(C)**, which could impact protein translation and could potentially contribute to the generation of non-canonical MHC-I binding peptides. These events could increase cross-dressing of dendritic cells by tumor cell derived MHC-I:peptide complexes and enhance activation of naïve antitumor CD8^+^ T cells and their differentiation towards CTLs. The ability of SOCS1 in activating p53 and senescence induction could potentially modulate p53-driven expression of NK cell ligands **(D)**, which could impact NK-cell mediated tumor cell killing. As a critical regulator of IFNγ, SOCS1 can inhibit adaptive immunosuppression mediated by IFNγ-induced PD-L1 expression in tumor cells **(E)**. Reported data are indicated by solid arrows. Proposed functions that need to be experimentally tested are indicated by dotted arrows. Figure created with Biorender.com.

Even though silencing SOCS1 in dendritic cells has been shown to enhance antigen presentation and antitumor immunity (66), SOCS1 may play an opposite role in cancer cells. Aside from binding and inhibiting JAKs and RTKs, SOCS1 functions as a substrate adaptor for protein ubiquitination that facilitates degradation by proteasomes (5, 6, 67). SOCS1 is known to interact with several signaling proteins via the central SH2 domain although other regions of SOCS1 may also be involved. The carboxy terminal of SOCS1 harbors the SOCS box domain, which is conserved among SOCS proteins and is composed of the BC box and a Cullin box. Whereas the Cullin box interacts with Cullin 5, the BC box interacts with elongin B and elongin C and brings in the RING-finger-domain-only protein RBX2 to assemble the Cullin5-RING-ubiquitin ligase CRL5^SOCS1^ (67–69). Others and we have shown that the ubiquitin-ligase function of SOCS1 promotes ubiquitination and proteasomal degradation of several oncogenic signaling proteins (5, 20, 52, 56, 70–76). Indeed, the list of signaling proteins regulated by SOCS1 is so diverse that the SOCS1-dependent proteasomal degradation may have a fundamental anti-tumor function that is coupled to inhibition of the oncogenic signaling pathways (**Figure 2A**).

In addition to binding proteins and targeting them for ubiquitination, SOCS1 has the potential to modulate both the cellular ubiquitination system and the proteasome composition. In a proteomic study using Hepa-vector and Hepa-SOCS1 cells, we observed that SOCS1 increased the expression of certain E2 Ub conjugation enzymes, which transfer the Ub moiety to the E3 ligase for eventual transfer to the substrate protein (77, 78). SOCS1 also caused a similar modification in the proteasome subunit composition (77, 79). These findings raise the possibility that SOCS1, induced by diverse growth stimuli, may also perform additional tasks besides attenuating signal transduction. These changes in ubiquitination and proteasome machinery, which may influence cellular protein homeostasis, can alter the quality and quantity of peptides generated by proteasomes (**Figure 2B**).

In normal and cancer cells, proteasomes play a crucial role in normal protein turn over as well as in degrading misfolded proteins (78, 80). This process generates peptides that are presented by MHC class-I molecules to facilitate cancer immune surveillance by CD8^+^ T lymphocytes (81–83). In the cancer immunity cycle, the initial activation naïve antitumor CD8^+^ T cells that recognize potential cancer antigens occurs when dendritic cells that take up tumor cell fragments process and present tumor antigens as MHC-I:peptide complex to naïve CD8^+^ T cells in tumor draining lymph nodes by a process called antigen cross-presentation (84–89). However, emerging evidence indicate that antigen presentation by ‘cross-dressing’ is the main activation mechanism of tumor-reactive CD8^+^ T cells. This process involves the uptake of MHC:peptide complexes from live tumor cells by professional antigen presenting cells via trogocytosis (90–94). We propose that by promoting ubiquitin-dependent proteasomal degradation, SOCS1 expression in tumor cells can potentially increase antigen processing and presentation and thereby increase the efficiency of the cross-dressing pathway and promote antitumor immunity (**Figure 2**).

### 4.2 Generation of non-canonical MHC-I peptides

The tumor immune surveillance concept postulates that the immune system is constantly on the lookout for cancer cells by recognizing abnormally expressed cellular proteins, which fall into two main groups (95–98): (i) tumor-specific neoantigens, which arise from mutations and give rise to antigenic peptides encompassing the mutated protein sequence that were never encountered by the immune system (99); (ii) tumor-associated antigens, which arise from aberrant expression of embryonic proteins due to the dedifferentiation process associated with tumor progression, and thus can elicit immune responses (100). With the advent of genome sequencing, identification of tumor neoantigens arising from mutations in a tumor specimen has become a manageable task, and this has led to the development of tumor vaccines based on neo-antigenic peptides and CAR-T cells reactive to them for cellular therapy (99, 101). These approaches have demonstrated feasibility and success, but arguments highlighting the limitations of this approach are also raised (102–106). Firstly, in order to be presented to CD8^+^ T cells, the mutant peptide must reside within an amino acid sequence that would permit binding to MHC-I. Secondly, generation of MHC-I binding peptides at steady state is coupled to protein biosynthesis and this process is estimated to be limited in efficiency, i.e out of two million peptides generated per second only 150 peptides get loaded onto MHC-I, likely dictated by the ability to bind the peptide binding grove of MHC-I (107). Third, it has been recognized for a long time that MHC-I associated peptides can arise from non-canonical protein sequences, including altered reading frames (defective ribosomal products, DRiPs) and non-coding regions of the messenger RNA and RNAs arising from intronic sequences (108–111). Such peptides would not be present in the canonical, annotated protein coding sequence databases used to identify mutations, which represents only 2% of the genome. In fact, only a small fraction of the MHC-I immunopeptidome is derived from protein-coding sequences (112). Mass spectrometry analysis of peptides eluted from MHC-I molecules of human cancers have revealed that non-canonical MHC-I peptides are far more abundant than mutated peptides and they originate from chromosomal regions that are distinct from mutational hotspots and not shared by embryonic cells (111, 113–115). Immunization with a candidate non-canonical peptide has been shown to elicit protective antitumor immunity (116). Even though current approaches to identify the non-canonical MHC-I peptides rely on mass spectrometry-based detection of peptides eluted from MHC-I molecules, approaches involving generation of databases containing transcriptomes of the entire genome and predicted proteomes of six reading frames (three on each DNA strand) are being employed in a proteogenomic approach to predict potential non-canonical MHC-I peptides (117, 118).

We have observed that Hepa-SOCS1 cells displayed marked alterations in the constituents of spliceosome, a multiprotein complex involved in processing RNA transcripts to mediate splicing, remove intronic sequences and generate mRNA for translation (77). In a recent study using shotgun proteomics employing data-independent acquisition, we observed that SOCS1-expressing Hepa cells showed upregulation of several hundred proteins (65). Pathway analysis of significantly upregulated proteins revealed that these proteins are enriched for the biological processes related to RNA splicing, mRNA processing, non-coding RNA (ncRNA) processing, rRNA processing and ribonucleoprotein complex biogenesis (**Figure 1E**), and showed significant enrichment within the cellular compartments of spliceosome, U2-spliceosome, catalytic step 2 spliceosome, nuclear speck and chromosomal region (**Figure 1F**), suggesting a profound impact of SOCS1 on pre-mRNA processing that could generate alternate splice forms. In addition, SOCS1-expressing Hepa cells displayed significant upregulation of protein subunits of ribosome and ribonucleoprotein complex (**Figures 1E, F**), suggesting potential impact of SOCS1 on protein translation as well. Moreover, non-canonical MHC-I peptides can arise from fusion of unrelated peptides during proteasomal processing (119, 120). Whether the marked alterations in the composition of proteasome subunits in SOCS1 expressing cells impact the generation of proteasome-spliced peptides that bind MHC-I needs to be tested. These postulated alterations in RNA processing, their translation to polypeptides and proteasomal degradation promoted by SOCS1 could promote the generation of non-canonical antigenic peptides (**Figure 2C**), which in turn would impact the MHC-I peptidome, potentially contributing to tumor immune surveillance. As SOCS1 expression at steady state is very low and is highly induced by mitogenic cytokines, growth factors and oncogenic growth signaling (6, 8), SOCS1 is very well poised to play a key role tumor immune surveillance. This could be an important factor determining the widespread repression of the *SOCS1* gene in diverse tumors.

### 4.3 Immune ligand expression by p53

Another potential influence of SOCS1 in modulating antitumor immune responses could occur via activating p53. In a cellular senescence model, oncogene-induced SOCS1 promotes p53 activation to induce senescence associated genes (59, 121). The p53 tumor suppressor plays multiple roles in mediating its functions. Depending on the extent of DNA damage, p53 facilitates DNA damage repair, or induce senescence or apoptosis to control tumor growth (122). The p53-induced senescence is also associated with the induction of natural killer (NK) cell activation ligands (RAE1, MULT1/ULBP1, H60A), chemokines and cytokines (CSF1, CCL2, CXCL1, IL-15), which can impact antitumor immune responses (123–126). In addition to CD8^+^ T cells, which play a key role in tumor immune surveillance, NK cells also play important roles in tumor control and tumor immune surveillance (127–130). As SOCS1 also harbors a nuclear localization signal and is required for activating p53-dependent senescence (59, 121, 131), it is not unlikely that SOCS1 may promote the induction of p53-dependent immune function-related genes and contribute to immune cell-mediated tumor control by inducing NK cell ligands and chemokines (**Figure 2D**).

### 4.4 Inhibition of immune checkpoint ligand expression

Activated, cytotoxic CD8^+^ T cells generated in draining lymph nodes emigrate, enter circulation, infiltrate tumors, engage tumor cells expressing cognate tumor antigenic peptide and release their cytotoxic granules to kill tumors (84, 132). At the same time these cytotoxic T lymphocytes also release effector cytokines, including IFNγ. IFNγ is a potent stimulator of the checkpoint ligand PD-L1, which engages PD1 on activated T cells and delivers an inhibitory signal to dampen their effector functions (133–135). Indeed, this adaptive immunosuppression mediated by IFNγ-induced PD-L1 expression is an important obstacle to efficient immune cell mediated tumor killing as it can inhibit cytotoxic CD8^+^ T and NK cells (136–139), although PD-L1 expression in tumors is considered a predictive biomarker for the effectiveness of immune checkpoint inhibitor therapy (140, 141). Given that SOCS1 is a non-redundant inhibitor of IFNγ signaling (7, 8), SOCS1 expression in cancer cells would dampen PD-L1-mediated checkpoint inhibition. Indeed, Naka and colleagues have shown that intra-tumoral delivery of SOCS1 by adenoviral vectors resulted in reduced PD-L1 expression that was correlated increased CD8^+^ T cell activation and improved tumor growth control (142). Thus, relieving the blockade on IFNγ signaling that upregulates the checkpoint ligand (**Figure 2E**) could be another reason why cancer cells choose to repress SOCS1 expression by diverse mechanisms.

## 5 Restoring SOCS1 expression in cancers

The cell-intrinsic tumor suppressor functions of SOCS1 and its potential contribution to antitumor immune responses raised the possibility of restoring SOCS1 expression to control tumor growth. The SOCS1 mimetic peptide Tkip has been shown to inhibit prostate cancer growth *in vitro* (55), indicating the potential utility of SOCS1-mediated inhibition in tumor growth control. However, it will be a challenging task to introduce SOCS1 mimetic peptides into all cancer cells. Moreover, such peptide mimetics that inhibit JAK kinases are unlikely to mediate the complex functions of SOCS1 in mediating protein degradation, activating p53, or induing the expression of several genes involved in RNA splicing and translation discussed above. SOCS1 gene therapy using adenoviral vectors in experimental models has been reported to reduce tumor growth by inhibiting oncogenic signaling and improving CD8^+^ T cell responses (142). As SOCS1 expression is highly regulated (6), stable constitutive expression of SOCS1 may lead to unintended consequences. As the *SOCS1* gene repression mainly occurs via CpG methylation in diverse cancers, demethylating agents such as 5-azacytidine and decitabine could be potentially useful to restore regulated SOCS1 expression (41, 143). Even though demethylating agents do have off-target effects, 5-aza is an already approved anticancer drug (144, 145). SOCS1 inhibition by miR155 also occurs commonly in several cancers (46, 146, 147). Even though miR-155 sponges and miR-155 targeting oligos can be used to restore SOCS1 expression *in vitro* settings (148, 149), miR-155 targets many signaling molecules (150) and the effectiveness of such approaches *in vivo* could be challenging. Mouse cancer models with *Socs1* gene repression remain scanty. Development of such models, also expressing surrogate tumor antigens, would help test the effectiveness of restoring endogenous SOCS1 expression and testing its impact on antitumor immune responses.

## 6 Discussion

The role of SOCS1 as a tumor suppressor has been well established and some of the underlying mechanisms include attenuation of mitogenic cytokine and growth factor signaling via the JAK-STAT and RTK pathways, inhibition of oncogenic signaling proteins by promoting their ubiquitination and proteasomal degradation, prevention of the oncogenic potential of tumor suppressors such as p21 and inhibition of NRF2-mediated tumor cell adaptation to elevated oxidative stress associated with neoplastic growth. Here, we have presented evidence that support potential cell-extrinsic role of SOCS1 in facilitating tumor immune surveillance and immune cell mediated tumor growth control via promoting tumor antigen processing and presentation, generation of non-canonical MHC-I peptides, expression of immune cell ligands and inhibition of immune checkpoint ligand expression (**Figure 2**). Validating these hypotheses will provide a compelling argument for the use of epigenetic modifying drugs to reverse SOCS1 gene repression to restore cell-intrinsic tumor suppressor functions as well as cell-extrinsic impact on antitumor immunity.

## Data availability statement

The original contributions presented in the study are included in the article/supplementary materials, further inquiries can be directed to the corresponding author/s.

## Ethics statement

The animal study was approved by Université de Sherbrooke Ethics Committee for Animal Care and Use. The study was conducted in accordance with the local legislation and institutional requirements.

## Author contributions

SI: Conceptualization, Data curation, Formal Analysis, Funding acquisition, Investigation, Supervision, Writing – original draft, Writing – review & editing. YG: Methodology, Writing – review & editing, Data curation, Formal Analysis, Investigation. AS: Data curation, Formal Analysis, Methodology, Writing – review & editing, Investigation. SR: Conceptualization, Resources, Writing – review & editing.
